# Highly clonal carbapenem-resistant *Acinetobacter baumannii* isolates from a Peruvian hospital

**DOI:** 10.1128/spectrum.02119-25

**Published:** 2025-10-20

**Authors:** Maria J. Pons, Fernando Guibert, Jorge Choque-Matos, Luciano A. Palomino-Kobayashi, Paul Fernandez-Castro, Patricia Gonzales, Carmen Valera-Krumdieck, Nidia Vilar, Carla Andrea Alonso, María López, Beatriz Rojo-Bezares, Yolanda Sáenz, Joaquim Ruiz

**Affiliations:** 1 Grupo de Investigación en Dinámicas y Epidemiología de la Resistencia a Antimicrobianos – “One Health”, Universidad Científica del Sur, Lima, Peru; 2 Grupo de Medicina Regenerativa, Universidad Científica del Sur, Lima, Peru; 3 Instituto Nacional de Salud 54719, Lima, Peru; 4 Instituto de Investigación en Ganadería y Biotecnología, Universidad Nacional Toribio Rodríguez de Mendoza, Amazonas, Peru; 5 Servicio de Enfermedades Infecciosas, Hospital Maria Auxiliadora, Lima, Peru; 6 Servicio es de Patología Clínica área de Microbiología del Hospital María Auxiliadora, Lima, Peru; 7 Área de Microbiología Molecular, Centro de Investigación Biomédica de La Rioja 541787, Logroño, Spain; 8 Departamento de Diagnóstico Biomédico, Laboratorio de Microbiología, Hospital Universitario San Pedro, Logroño, Spain; The University of Sydney, Darlington, Australia

**Keywords:** *Acinetobacter baumannii*, carbapenem resistance, carbapenemases, healthcare-associated infections, clonal relationship, South America

## Abstract

*Acinetobacter baumannii* is an opportunistic pathogen of great clinical concern, showing high levels of resistance to several antimicrobials, including carbapenems. This study aimed to characterize carbapenem-resistant *A. baumannii* recovered from a tertiary care hospital (Lima, Peru) in January–February 2021 (during the coronavirus disease 2019 pandemic). Antimicrobial susceptibility was determined by VITEK-2, and the presence of carbapenem-resistance genes by PCR. All 40 *A*. *baumannii* isolates were extensively drug-resistant, showing full resistance to all antibiotics tested except ampicillin/sulbactam (95% of resistance) and colistin (10%). The *bla*_OXA-24-like_ gene was detected in 90% of isolates, and the remaining 10% isolates harbored the *bla*_OXA-23-like_ gene. Pulsed-field gel electrophoresis classified isolates into three different pulsotypes, grouping 8, 2, and 30 isolates, respectively. Additionally, whole-genome sequencing of two isolates of the main pulsotype revealed that they belonged to ST2 and ST2724 (international clone II) and harbored the *bla*_TEM-1_, *bla*_OXA-66_, *bla*_OXA-72_, and *bla*_ADC-30_ β-lactamase genes; mutations in *gyrA* and *parC*, aminoglycoside, tetracycline, and sulfonamide resistance genes; and a class 1 integron (In439) containing *aac(6′)-Ib′*, *catB8*, and *aadA1* located inside the AbGRI3 genomic resistance island. These findings highlight the presence of highly resistant and clonally related ST2 of *A. baumannii* and their relationship with genomes of ST2 of South America.

**IMPORTANCE:**

*Acinetobacter baumannii* is a critical pathogen known for causing severe infections in hospitalized patients and for its remarkable ability to develop resistance to multiple antibiotics, including last-line treatments such as carbapenems. In Latin America, data on the genetic background and spread of these resistant strains remain limited. Our study provides a detailed molecular and genomic characterization of extensively drug-resistant *A. baumannii* isolates collected from a Peruvian hospital during the coronavirus disease 2019 pandemic. We identified a predominant clone belonging to international clone II, which carried multiple resistance genes and a complex resistance island. These findings raise public health concerns, as they highlight the silent spread of high-risk clones in regions with limited surveillance capacity. By contributing valuable local genomic data, our study supports global efforts to track antimicrobial resistance and underscores the urgent need to strengthen molecular monitoring of hospital-associated pathogens also in low- and middle-income countries.

## INTRODUCTION

*Acinetobacter baumannii* is an opportunistic pathogen responsible for a wide range of infections and is one of the most frequent microorganisms causing healthcare-associated infections (1). In addition, the intensive use of antimicrobials, especially in intensive care units (ICUs), has contributed to the natural selection of antimicrobial-resistant pathogens, with frequent outbreaks of highly resistant strains worldwide (2, 3). Furthermore, the severe acute respiratory syndrome coronavirus 2 pandemic (coronavirus disease 2019 [COVID-19]) increased the presence of resistant bacteria, leading to increased difficulty in the treatment, management, and control measures of patients infected with extremely drug-resistant (XDR) and/or pan drug-resistant (PDR) isolates (4).

*A. baumannii* has developed resistance to most of the antibiotics currently available; thus, in recent years, levels of antimicrobial resistance increasing at a worrying rate, including resistance to antibiotics such as carbapenems or colistin (5, 6). In this sense, the World Health Organization has included carbapenem-resistant *A. baumannii* in the “critical group” of bacterial pathogens because of its common XDR phenotype (including resistance to last-resort antibacterial agents), its ability to horizontally transfer resistance mechanisms, and the severity of the resulting infections, which altogether result in a major threat to human health (7).

The carbapenem resistance of *A. baumannii* may be related to a variety of carbapenemases, which are primarily related to acquired class D (OXA-type) carbapenemases (8, 9). Among these, the most commonly described worldwide are those belonging to the OXA-23 and OXA-24 groups, with the OXA-23 group being the most frequently described, and the OXA-24 group showing a high frequency among North and South American isolates (9).

It has been observed that the burden of healthcare-associated infections is higher in resource-limited countries (10), highlighting the potential impact of the high antimicrobial resistance of clinically important bacterial infections on the health systems and livelihoods of these regions that already suffer from broader socioeconomic problems and further hinder the control of these infections (11).

It is necessary to understand the dynamics of antimicrobial resistance at regional and local levels in order to design mitigation strategies in accordance with the therapeutic options available in the corresponding health systems (12). In countries such as Peru, studies on the characterization of *A. baumannii* have increased in recent years (3, 13–15), but it is necessary to update these studies and determine the impact of the COVID pandemic on this microorganism, both in the country and in the region.

In this scenario, this study aimed to characterize a series of *A. baumannii* isolates related to human infections during the COVID-19 pandemic in Peru.

## MATERIALS AND METHODS

### Bacterial isolates

This study included 40 consecutive non-redundant clinical *A. baumannii* isolates collected from 40 different inpatients at a tertiary care hospital (Hospital Maria Auxiliadora) in Lima (Peru) during January–February 2021. The microorganisms were obtained from different types of samples: blood, respiratory samples, and soft tissues. The isolates were then identified as *A. baumannii* using the VITEK-2 (bioMérieux) automated bacterial identification system and confirmed by matrix-assisted laser desorption/ionization time-of-flight using a MALDI Biotyper (Bruker Daltonics GmbH & Co. KG, Bremen, Germany) with the MBT Compass Library DB-6903 (V.6) (May 2016).

### Antimicrobial susceptibility

Susceptibility to 12 antibiotics, including ampicillin/sulbactam, piperacillin/tazobactam, ceftazidime, cefepime, cefotaxime, imipenem, meropenem, gentamicin, amikacin, ciprofloxacin, and trimethoprim/sulfamethoxazole, was determined using the VITEK-2 automated system, as well as the Kirby-Bauer disk diffusion method, following the guidelines of the Clinical and Laboratory Standards Institute (16). Meanwhile, colistin resistance was established following previously described procedures (17). Resistant and intermediate isolates were categorized together for analysis purposes as “non-susceptible.” Isolates resistant to all antibacterial families tested, but two were defined as XDR, while potential PDR isolates were those exhibiting resistance to all tested antimicrobial agents.

### Carbapenemase characterization

The presence of genes encoding the following serine-type carbapenemases was determined by polymerase chain reaction (PCR): class A (GES, KPC, and IMI) and class D (OXA-23 group, OXA-24 group, OXA-48 group, and OXA-58 group), as well as metallo-β-lactamases (IMP, VIM, and NDM) (18, 19). Additionally, the presence of the *bla*_PER_ gene, coding for an extended-spectrum β-lactamase, was also analyzed by PCR (18).

### Pulsed-field gel electrophoresis

Pulsed-field gel electrophoresis (PFGE) was performed by digesting genomic DNA with the *Apa*I restriction enzyme and a CHEF-DRIII system (Bio-Rad Laboratories, Hercules, CA, USA), following the modified protocol of Gautom (20). Briefly, the agarose plugs were prepared from a bacterial suspension in SE buffer (75 mM NaCl, pH 8.0; 25 mM EDTA, pH 8.0) adjusted to an optical density of 1.6–1.7 at a wavelength of 610 nm. After lysis and washing, the plugs were digested with 10 U of *Apa*I enzyme (New England, Biolabs) according to the manufacturer’s instructions. The DNA fragments were resolved in 1% agarose gels using a CHEF-DRIII system under the following conditions: 6 V/cm, 14°C, and pulse time ranging from 0.5 s to 15 s during 19 h. PFGE patterns were analyzed using the GelJ v2.0 software (21). The cluster analysis was done by the unweighted pair group method using arithmetic mean and the Dice similarity coefficient, with a 1.0% band position tolerance. An identity of ≥90% was considered to classify isolates as indistinguishable, and ≥80% as belonging to the same epidemiological clonal group (3, 22).

### Whole-genome sequencing

Two representative isolates of the most frequent clone were selected for whole-genome sequencing. Genomic DNA was extracted and purified using the PureLink Genomic DNA Mini Kit (Invitrogen, Waltham, MA, USA), following the manufacturer’s instructions. DNA concentration and purity were assessed with a spectrophotometer (Denovix, Wilmington, USA). Finally, the concentration was measured by the fluorometric method using the Qubit 3.0 fluorometer (Invitrogen, Malaysia) and Qubit dsDNA BR Assay Kit. DNA was stored at −20°C until use. Libraries were made using the Nextera XT Library Prep Kit (Illumina, San Diego, USA). Genomic sequencing was performed using the MiSeq Reagent Kit v3 (600 cycles) and the Illumina Miseq platform (Illumina, San Diego, CA, USA) with a paired-end read chemistry (2 × 300 bp) at the Laboratory of Molecular Biology and Biotechnology of the National Institute of Health, Peru. Quality control (QC) of reads was evaluated using FastQC v0.12.1 (23), while completeness and contamination levels were predicted by CheckM2 v1.1.0 (24). Low-quality adapters and nitrogenous bases were removed using Trimmomatic v0.39 (25). *De novo* genome assembly was carried out using SPAdes v3.15.15 (26) and IDBA v1.1.3 (27). QC of assemblies was analyzed with QUAST 5.2.0 (28). Average nucleotide identity (ANI) was obtained with the ANI calculator (29). Gene annotation was performed using Prokka v1.14.5 (30). Multi-locus sequence typing (MLST) alleles, using the Pasteur scheme, and sequence types were obtained using MLST v2.23.0.(https://github.com/tseemann/mlst). *In silico* antigen typing was done using Kaptive v3.1.0 (31). Plasmid replicons were identified by BLAST using the *Acinetobacter* Typing database (https://github.com/MehradHamidian/AcinetobacterPlasmidTyping) (32).

All the publicly available genomes from *A. baumannii* ST2 or ST2724 (closely related to ST2) from the South American continent, as well as the corresponding metadata in .tsv format, were downloaded from the NCBI Isolates browser (https://www.ncbi.nlm.nih.gov/pathogens/isolates) for assessment of phylogenetic comparisons. Very fragmented genomes (>500 contigs) or genomes with contamination levels of more than 5% were excluded. With these considerations, 96 genomes were included in the following analyses. Assembly metrics, MLST determination, and gene annotation for these genomes were done as stated above. A pangenome was established using Roary v3.13 (33). Informative-only sites from the core-genome alignment were extracted using SNP-sites v2.5.1, and a maximum-likelihood tree was constructed using IQ-TREE v2.3.6 (34), with automatic model selection and ultrafast bootstrap options enabled (35, 36). The graphic representation of the tree was done in R v4.4.2 using the package ggtree.

### Predicted antimicrobial resistance determinants

Identification of predicted antimicrobial resistance determinants was obtained using AMRfinderplus v3.11.20 within the AMRColab tool (37), requiring the draft-genome sequences in fasta format as input.

## RESULTS

### Study population and bacterial isolates

**Fig 1:**
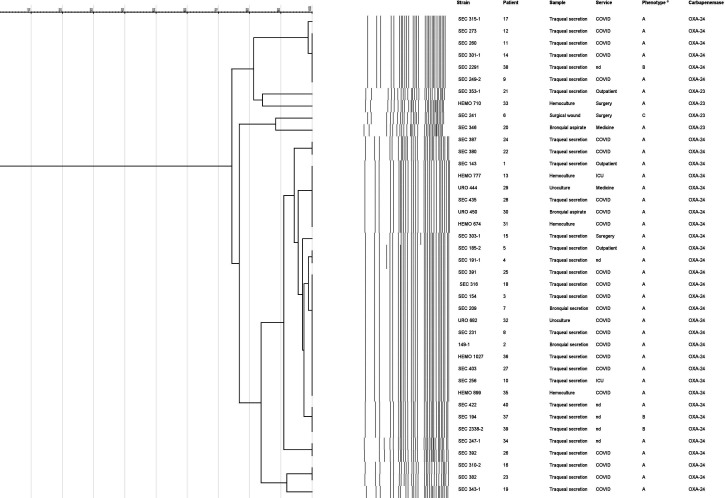
PFGE patterns and characteristics of the 40 *A*. *baumannii* isolates. nd, non-determined. ªPhenotype A: all isolates were resistant to piperacillin/tazobactam, ceftazidime, ampicillin/sulbactam, cefepime, cefotaxime, imipenem, meropenem, ciprofloxacin, trimethoprim/sulfamethoxazole, gentamicin, and amikacin. Phenotype B: Phenotype A plus colistin resistance. Phenotype C: Phenotype B, but susceptible to ampicillin/sulbactam.

Forty *A. baumannii* isolates were included in the study. Apart from 6 isolates that had no related epidemiological data, the information from the remaining 34 isolates showed that the mean age of the patients was 50.5 ± 13.3 years and only 6 (16.7 %) were female. Twenty-four out of 34 isolates (70.1%) were from COVID-19 patients, and the others were from outpatients, ICU, surgery, and medicine units (Fig. 1). Of the 40 isolates, 82.5% were recovered from respiratory samples, followed by hematological samples (10%), while 5% were from urine samples and one isolate (2.5%) from a wound sample (Fig. 1).

### Antimicrobial susceptibility

All *A. baumannii* isolates were resistant to piperacillin/tazobactam, ceftazidime, cefepime, cefotaxime, imipenem, meropenem, ciprofloxacin, trimethoprim/sulfamethoxazole, gentamicin, and amikacin. Resistance values were also high for ampicillin/sulbactam (95%). Remarkably, four isolates were colistin-resistant (10%) (Fig. 1). All isolates exhibited an XDR phenotype, except for three (7.5%) that were PDR.

### Carbapenemases

The *bla*_OXA-24-like_ was the most frequently detected gene, being observed in 36 isolates (90%), and the remaining four isolates harbored the *bla*_OXA-23-like_ gene (10%) (Fig. 1). None of the other searched genes was detected.

### Pulsed-field gel electrophoresis

PFGE (identity > 80%) allowed the classification of all *A. baumannii* isolates into three different pulsotypes (PI to PIII). Indeed, all 40 isolates had a homology >75%. Pulsotype III was identified in 75% of the isolates (which included isolates of different origins: outpatients, COVID, ICU, medicine, and surgery units) (Fig. 1).

### Whole-genome sequence

Two isolates, HEMO-777 and SEC-154, of the main pulsotype (PIII) were whole-genome sequenced (NCBI Biosamples SAMN42375209 and SAMN42035152, respectively), and their genome data have been deposited at NCBI using BioProject number PRJNA1128084, and the genome (nucleotide) GenBank accession numbers GCA_051942065 (JBFECW000000000) and GCA_051941745 (JBEUKO000000000), respectively.

The genomic features of both isolates are summarized in Table 1. Both genomes carried the same antimicrobial resistance determinants, including *bla*_TEM1_, *bla*_OXA-72_, and the chromosomal *bla*_OXA-66_ and AmpC β-lactamase *bla*_ADC-30_, as well as point mutations in *gyrA* and *parC*. In addition, several acquired resistance elements were found. These included aminoglycoside, tetracycline, and sulfonamide resistance genes, and a class 1 integron (In439, named by Integrall database http://integrall.bio.ua.pt) carrying the *aac(6′)-Ib′*, *catB8*, and *aadA1* gene cassettes. This integron was located within the genomic resistance island AbGRI3-2i, which also harbors the *armA*, *msr(E*), and *mph(E*) genes (Table 1). Also, the insertion of ISAba1 sequences upstream of *bla*ampC was detected.

**TABLE 1:** Antibiotic and disinfectant resistance genes detected in whole-genome sequencing of two *A. baumannii* isolates

Antibiotics	Antibiotic resistance mechanisms
Quinolones	GyrA_S81L, ParC_S84L
Penicillins, cephalosporins	*bla*_ADC-30_ ^*a*^
Penicillins, carbapenems	*bla*_OXA-66_ (OXA-51 family-intrinsic)^*a*^
Carbapenems	*bla*_OXA-72_ (OXA-24 family-acquired)
Penicillins, narrow spectrum cephalosporins	*bla*_TEM-1_
Aminoglycosides	*aph(3″)-Ib (strA)*, *ant(3″)-IIa (aadB)*, *aadA1*, *aph(6)-Id (strB)*, *aac(6′)-Ian*, *aph(3′)-Ia (aphA1)*, *aac(6′)-Ib (aacA4*), *armA*
Sulfonamides	*sul1*, *sul2*
Tetracycline	*tetA*, *tetB*
Fosfomycin	*abaF* ^*a*^
Phenicol (chloramphenicol)	*catB8*
Macrolide	*msr(E)*, *mph(E*)
Multidrug efflux MFS transporter	*amvA* ^*a*^
Multidrug efflux RND transporter	*adeABC* ^*a*^
Nickel	*nreB*
Quaternary ammonium compounds (QACs)	*qacE*Δ*1*

^*a*^ Encoded within bacterial chromosome. Must be overexpressed to display a resistant phenotype.

Additionally, the assignment of the capsule (KL) and lipooligosaccharide (OCL) loci in the two sequenced *A. baumannii* genomes was identified as KL9 at the capsular locus and OCL1 at the outer core lipooligosaccharide locus. Also, replicon plasmid analysis showed that both belong to r3-T3. Concerning the MLST (Pasteur scheme), one of the two isolates sequenced belonged to ST2, while the other was a new ST (ST2724) very similar to ST2, except in the *cpn60* gene, which presented variations registered as allele 509.

**Fig 2:**
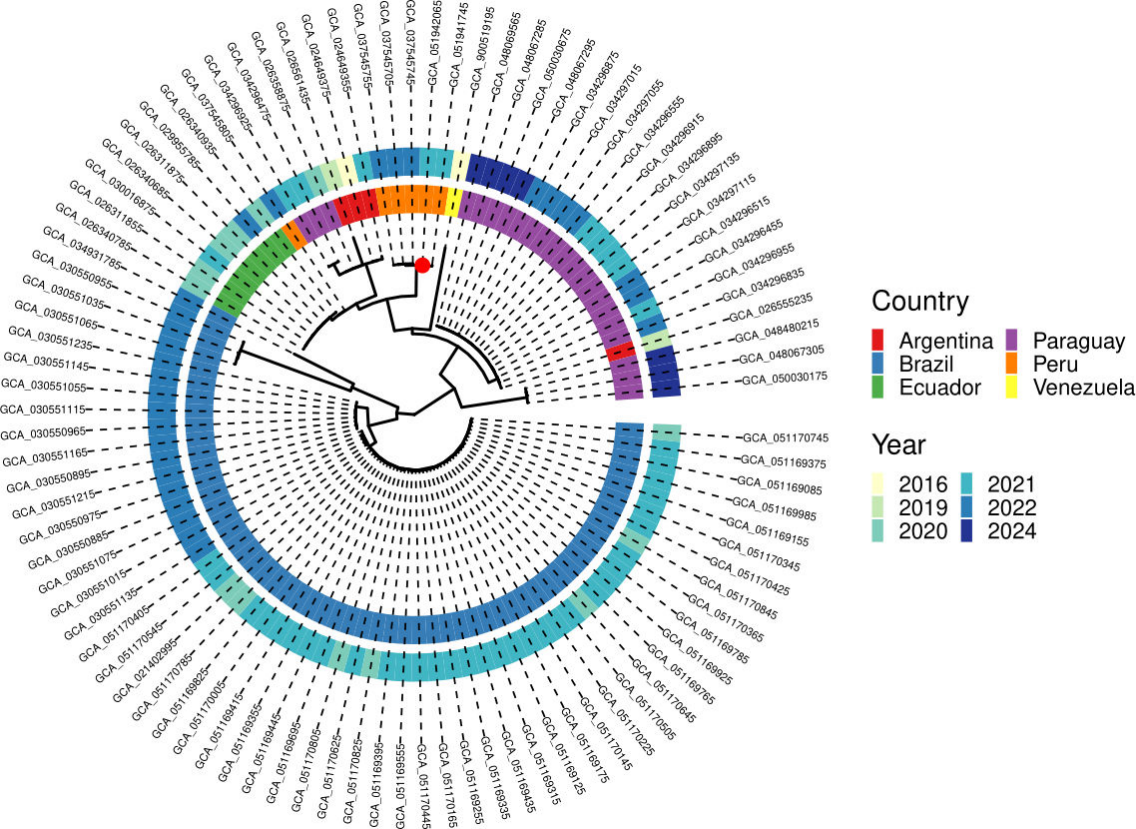
Midpoint-rooted maximum-likelihood phylogenetic tree of *A. baumannii* ST2 and ST2724 genomes from South America. The innermost ring-colored tips show country of isolation, whereas the outer ring of colored boxes indicates year of isolation. The two Peruvian isolates from our study (GCA_051942065.1 and GCA_051941745.1) are indicated by a red dot.

To put our strains in a regional context, our genomes were compared with *A. baumannii* ST2 and ST2724 genomes from South America available in GenBank. The phylogenetic tree showed well-supported clades (nodes with high bootstrap values), and our strains were grouped in a subclade with 100/100 support with other Peruvian strains isolated from Ayacucho in 2022 and 2024. Within the closest clade, additional Peruvian strains from the Amazonas region reported in 2022 were identified, as well as Ecuadorian strains from the same year. *A. baumannii* isolates from Argentina and Paraguay in the years 2016–2023 were also in a close clade. It should be noted that Brazil reported more than half of the available strains of *A. baumannii* ST2 since the beginning of 2019 (Fig. 2).

## DISCUSSION

There are few studies on the epidemiology and characterization of carbapenemases of *A. baumannii* in Latin America, and those available are mainly focused on the clonality approach (3, 38, 39).

The rise in resistance of this pathogen is a matter of concern, given the increasing number of XDR *A. baumannii* isolates reported in recent years (6). In Latin America, a 5-year study demonstrated that *A. baumannii* isolates became resistant to certain antibiotics, such as cephalosporins and carbapenems (40). Furthermore, we have also observed the emergence of highly concerning PDR strains. Both XDR and PDR *A. baumannii* isolates are linked to the rapid dissemination of a few epidemic lineages, such as ST2 (41). It is also necessary to identify these clones in low- and middle-income countries where genomic surveillance associated with health systems is weakened.

*A. baumannii* is a pathogen mostly associated with ventilator-associated pneumonia and bacteremia. This fact makes it a problem in the ICUs of many hospitals, with higher incidence rates that peaked in the COVID-ICU setting, as has been reported (42, 43). Of note, patients with COVID-19 and a bacterial coinfection present worse outcomes compared to patients without COVID-19 (44). These observations underscore the importance of active surveillance.

The levels of antibiotic resistance described in the present study are of great concern, with findings of 100% resistance to cephalosporins, carbapenems, quinolones, piperacillin-tazobactam, and aminoglycosides, and 95% resistance to ampicillin/sulbactam, leaving colistin as practically the only option for treatment. These antibiotic-resistant levels are higher than previously reported in Peru (3). While no data were recovered about antibiotic use, the COVID-19 pandemic resulted in a high use of antibiotics in health facilities, which, together with the extreme stress, patients exceeding hospital facility numbers, and a forced relaxation of hygiene measures, likely contributed to both the selection of almost untreatable *A. baumannii*, as well as to its spread throughout hospital facilities (45). Regarding colistin, the isolation of four resistant *A. baumannii* strains is of concern. Moreover, three of these strains could be classified as potential PDR strains, as they were resistant to all antibiotics tested in this study. Along this line, recent reports highlight the increasing prevalence of colistin resistance in *Acinetobacter* spp. (46). Colistin is considered a last-resort therapeutic option for carbapenem-resistant *A. baumannii*. With the emergence of colistin resistance in this pathogen, the therapeutic armamentarium available to treat *A. baumannii* infections has become very limited (46). This fact indicates the need to search for new treatments or new combinations of known antimicrobials, such as high-dose ampicillin/sulbactam, meropenem, plus polymyxin B (SUL/MEM/PMB) (47), eravacycline (48), or cefiderocol, a novel siderophore cephalosporin, which has displayed activity against carbapenem-resistant *A. baumannii* (49, 50). This drug, however, is not yet commercially available in most Latin American countries.

The increase and spread of carbapenem-resistant *A. baumannii* are mainly due to OXA-type acquired carbapenemases, although other carbapenemases, such as NDM, have been also described in clinical isolates in Peru (51). In the present study, *bla*_OXA-24-like_ was the most frequent carbapenemase-encoding gene detected. This result agrees with previous studies in the country (3, 15, 52), as well as with the reported high prevalence of *bla*_OXA-24-like_ in other South American countries (9). Notwithstanding, in some Peruvian health centers, *bla*_OXA-23-like_ has been found to be the most prevalent carbapenemase gene among *A. baumannii*, rather than *bla*_OXA-24-like_ (53). In recent years, an increase in the number of *A. baumannii* isolates showing co-production of different carbapenemases has been reported, especially that regarding the production of *bla*_OXA-23-like_ and *bla*_OXA-24-like_ genes (54).

Whole-genome sequencing of two isolates belonging to the most common pulsotype identified the *bla*_OXA-24-like_ gene as *bla*_OXA-72_. Surveillance of *A. baumannii* strains is recommended, as PDR strains of different clones carrying a plasmid containing the *bla*_OXA-72_ gene have been detected in the region (55, 56). Whole-genome sequencing also identified the presence of *bla*_OXA-66_, a specific variant of the intrinsic *bla*_OXA-51_ gene. In addition to carbapenemase genes, other genes or chromosomal mutations involved in the development of antimicrobial resistance (quinolones, aminoglycosides, penicillins, sulfonamides, tetracyclines, fosfomycins, phenicols, and macrolides) were found. Specifically, the *sul1*, *sul2*, *tetA*, and *tetB* genes have been previously identified and reported, potentially compromising treatment with sulfonamides and tetracyclines. It should be noted that ISAba1 was identified immediately upstream of the *bla*ampC gene, as described previously, providing promoter sequences that enhance high-level expression of the β-lactamase gene related to increased level of β-lactam resistance (57).

The presence of *qacEΔ1* was also detected, related to resistance to quaternary ammonium-type disinfectants. Previous studies in *A. baumannii* have reported high prevalence rates of *qacEΔ1* through an efflux transporter (58). The *qacEΔ1* gene was detected in this work as a part of the class 1 integron In439. This integron was located inside a genomic island described as AbGRI3-2i, which was previously detected in *A. baumannii* and also associated with international clone II (ICII) (59).

In addition to these resistance determinants, the capsular locus KL9 and the external core locus of lipooligosaccharide OCL1 matched the most common profiles described in international high-risk lineages, particularly in the ST2/IC2 clonal complex, which is widely spread globally (60).

Prior to our study, only four ST2 genomes from Peru were available in public databases. Our contribution of two additional genomes expands this limited data set with isolates from Peru and relates them to other ST2-related isolates from South America. Interestingly, until a few years ago, the predominant lineages of *A. baumannii*, such as international clones II and III, reported in Latin America differed from those observed in other parts of the world. However, in recent years, XDR clones of *A. baumannii* have been primarily associated with ICII, as described in isolates from healthcare settings in Peru (3), hospitals in Rio de Janeiro (61), and subsequent outbreaks in Brazil (62). In agreement with these findings, our results showed that the most frequent pulsotype belonged to, or was closely related to, ST2, also classified within clonal complex 2 and ICII. Notably, as a representative of ICII, ST2 is currently the most globally disseminated carbapenem-resistant lineage and is frequently implicated in XDR outbreaks worldwide (63).

In addition, phylogenomic analysis showed the relevant location of our isolates in the same clade as isolates recovered from Peru (Ayacucho, in the Andean area approximately 550 km Southeast of Lima) in 2022. The clear separation of this Peruvian clade from other Latin American ST2 subclades suggests local persistence and dissemination of strains among healthcare facilities in the area. Moreover, the cluster is also closely related to previously reported ST2 genomes from the Amazonas region of Peru and from Ecuador, also suggesting regional dissemination of a specific subclade of this high-risk clone across the Andean–Amazonian area. Our study strengthens the genomic baseline for South America, enhances representation of Peruvian isolates in international databases, and provides critical data for future surveillance studies.

The limitations of this study include its development in a single center and a small sample size. Nevertheless, it provides valuable data on the molecular characteristics of high-risk *A. baumannii* isolates from Peru, a country where genomic information on this pathogen remains scarce. Additionally, the sampling period should be considered, as it took place during the COVID-19 pandemic, which may have influenced the distribution and characteristics of the *A. baumannii* population. However, this information is relevant for improving our understanding of the actual epidemiological situation in Peru, as well as a comparison with regional strains sharing the same ST during a period of extreme stress on healthcare facilities. Thus, this information could be helpful to assess the local epidemiology of *A. baumannii* at an extremely difficult time and thereby devise infection control strategies to prevent a similar scenario.

In summary, the present study shows the presence of clonally related highly antibiotic-resistant *A. baumannii* in a Peruvian setting, alerting about the risk of the appearance of PDR isolates. This study describes the antibiotic resistance present during the COVID-19 pandemic and demonstrates the need for genomic surveillance to determine the permanence and prevalence of these isolates in the area for correct calibration of the impact of the COVID-19 pandemic.
